# Supplementary material for: Modeling glioblastoma heterogeneity as a dynamic network of cell states
Source: Mol Syst Biol. 2021 Sep 16;17(9):e10105. doi: 10.15252/msb.202010105 (PMC8444284; doi:10.15252/msb.202010105)
Supplement: Supplementary file 6 — Source Data for Figure 5 [file MSB-17-e10105-s004.zip › Figure5A_sourcedata/GSEA_3071/nosigdb_stateVI.GseaPreranked.1622795169056/LARSSON_STATE_2.html]

Details for gene set LARSSON\_STATE\_2[GSEA]

|  || Dataset | state63071 |
| Phenotype | NoPhenotypeAvailable |
| Upregulated in class | na\_neg |
| GeneSet | LARSSON\_STATE\_2 |
| Enrichment Score (ES) | -0.6262989 |
| Normalized Enrichment Score (NES) | -2.7272985 |
| Nominal p-value | 0.0 |
| FDR q-value | 0.0 |
| FWER p-Value | 0.0 |
Table: GSEA Results Summary

  

Fig 1: Enrichment plot: LARSSON\_STATE\_2      
 Profile of the Running ES Score & Positions of GeneSet Members on the Rank Ordered List

  

| PROBE | GENE SYMBOL | GENE\_TITLE | RANK IN GENE LIST | RANK METRIC SCORE | RUNNING ES | CORE ENRICHMENT || 1 | HSPA5 |  |  | 414 | 0.484 | -0.1046 | No |
| 2 | PRDX4 |  |  | 647 | 0.412 | -0.1600 | No |
| 3 | LMAN1 |  |  | 1142 | 0.324 | -0.2905 | No |
| 4 | GPC1 |  |  | 1876 | -0.261 | -0.4891 | No |
| 5 | PDIA3 |  |  | 1879 | -0.261 | -0.4838 | No |
| 6 | PGRMC1 |  |  | 1889 | -0.262 | -0.4805 | No |
| 7 | LAPTM4A |  |  | 1898 | -0.263 | -0.4768 | No |
| 8 | ZNF75A |  |  | 1945 | -0.270 | -0.4835 | No |
| 9 | ATP6AP2 |  |  | 1974 | -0.274 | -0.4852 | No |
| 10 | LUC7L3 |  |  | 2072 | -0.290 | -0.5057 | No |
| 11 | MDM4 |  |  | 2188 | -0.313 | -0.5308 | No |
| 12 | NEAT1 |  |  | 2208 | -0.316 | -0.5290 | No |
| 13 | LENG8 |  |  | 2276 | -0.328 | -0.5403 | No |
| 14 | LRPAP1 |  |  | 2392 | -0.352 | -0.5644 | No |
| 15 | B2M |  |  | 2460 | -0.369 | -0.5748 | No |
| 16 | VMP1 |  |  | 2490 | -0.376 | -0.5745 | No |
| 17 | NKTR |  |  | 2536 | -0.385 | -0.5784 | No |
| 18 | CREBZF |  |  | 2542 | -0.387 | -0.5711 | No |
| 19 | ADGRV1 |  |  | 2741 | -0.437 | -0.6165 | Yes |
| 20 | SPPL2B |  |  | 2766 | -0.446 | -0.6131 | Yes |
| 21 | KANSL1 |  |  | 2800 | -0.459 | -0.6120 | Yes |
| 22 | FUS |  |  | 2845 | -0.474 | -0.6137 | Yes |
| 23 | PON2 |  |  | 2884 | -0.490 | -0.6132 | Yes |
| 24 | MIR99AHG |  |  | 2892 | -0.491 | -0.6041 | Yes |
| 25 | LAPTM4B |  |  | 2910 | -0.498 | -0.5977 | Yes |
| 26 | DDX17 |  |  | 2946 | -0.512 | -0.5959 | Yes |
| 27 | ARGLU1 |  |  | 2984 | -0.532 | -0.5943 | Yes |
| 28 | MARCH6 |  |  | 2985 | -0.532 | -0.5823 | Yes |
| 29 | RCN2 |  |  | 3090 | -0.580 | -0.5983 | Yes |
| 30 | XRCC2 |  |  | 3104 | -0.588 | -0.5887 | Yes |
| 31 | PRH1 |  |  | 3118 | -0.597 | -0.5789 | Yes |
| 32 | FTX |  |  | 3124 | -0.599 | -0.5669 | Yes |
| 33 | WSB1 |  |  | 3130 | -0.608 | -0.5546 | Yes |
| 34 | ZNF708 |  |  | 3214 | -0.684 | -0.5624 | Yes |
| 35 | MTRNR2L12 |  |  | 3251 | -0.713 | -0.5564 | Yes |
| 36 | KCNQ1OT1 |  |  | 3332 | -0.796 | -0.5608 | Yes |
| 37 | DDR1 |  |  | 3350 | -0.814 | -0.5472 | Yes |
| 38 | GRIA2 |  |  | 3351 | -0.815 | -0.5289 | Yes |
| 39 | PLAT |  |  | 3416 | -0.927 | -0.5259 | Yes |
| 40 | CD9 |  |  | 3445 | -0.995 | -0.5114 | Yes |
| 41 | APLP2 |  |  | 3446 | -1.001 | -0.4889 | Yes |
| 42 | SLC1A3 |  |  | 3458 | -1.025 | -0.4689 | Yes |
| 43 | FGFBP3 |  |  | 3502 | -1.120 | -0.4557 | Yes |
| 44 | NOVA1 |  |  | 3510 | -1.148 | -0.4318 | Yes |
| 45 | NCAM1 |  |  | 3514 | -1.168 | -0.4064 | Yes |
| 46 | GPM6A |  |  | 3515 | -1.169 | -0.3801 | Yes |
| 47 | SOX6 |  |  | 3517 | -1.174 | -0.3540 | Yes |
| 48 | APP |  |  | 3562 | -1.427 | -0.3342 | Yes |
| 49 | TNC |  |  | 3586 | -1.618 | -0.3042 | Yes |
| 50 | SCRG1 |  |  | 3613 | -2.165 | -0.2628 | Yes |
| 51 | GPM6B |  |  | 3616 | -2.257 | -0.2126 | Yes |
| 52 | SNTG1 |  |  | 3627 | -2.629 | -0.1563 | Yes |
| 53 | PTN |  |  | 3632 | -2.826 | -0.0939 | Yes |
| 54 | PTPRZ1 |  |  | 3637 | -4.237 | 0.0003 | Yes |
Table: GSEA details [plain text format]

  

Fig 2: LARSSON\_STATE\_2: Random ES distribution      
 Gene set null distribution of ES for **LARSSON\_STATE\_2**
